# Multiple Mitochondrial Introgression Events and Heteroplasmy in *Trypanosoma cruzi* Revealed by Maxicircle MLST and Next Generation Sequencing

**DOI:** 10.1371/journal.pntd.0001584

**Published:** 2012-04-10

**Authors:** Louisa A. Messenger, Martin S. Llewellyn, Tapan Bhattacharyya, Oscar Franzén, Michael D. Lewis, Juan David Ramírez, Hernan J. Carrasco, Björn Andersson, Michael A. Miles

**Affiliations:** 1 Department of Pathogen Molecular Biology, Faculty of Infectious and Tropical Diseases, London School of Hygiene and Tropical Medicine, London, United Kingdom; 2 Science for Life Laboratory, Department of Cell and Molecular Biology, Karolinska Institutet, Stockholm, Sweden; 3 Centro de Investigaciones en Microbiología y Parasitología Tropical, CIMPAT, Universidad de los Andes, Bogotá, Colombia; 4 Instituto de Medicina Tropical, Facultad de Medicina, Universidad Central de Venezuela, Los Chaguaramos, Caracas, Venezuela; Instituto Oswaldo Cruz, Fiocruz, Brazil

## Abstract

**Background:**

Mitochondrial DNA is a valuable taxonomic marker due to its relatively fast rate of evolution. In *Trypanosoma cruzi*, the causative agent of Chagas disease, the mitochondrial genome has a unique structural organization consisting of 20–50 maxicircles (∼20 kb) and thousands of minicircles (0.5–10 kb). *T. cruzi* is an early diverging protist displaying remarkable genetic heterogeneity and is recognized as a complex of six discrete typing units (DTUs). The majority of infected humans are asymptomatic for life while 30–35% develop potentially fatal cardiac and/or digestive syndromes. However, the relationship between specific clinical outcomes and *T. cruzi* genotype remains elusive. The availability of whole genome sequences has driven advances in high resolution genotyping techniques and re-invigorated interest in exploring the diversity present within the various DTUs.

**Methodology/Principal Findings:**

To describe intra-DTU diversity, we developed a highly resolutive maxicircle multilocus sequence typing (mtMLST) scheme based on ten gene fragments. A panel of 32 TcI isolates was genotyped using the mtMLST scheme, *GPI*, mini-exon and 25 microsatellite loci. Comparison of nuclear and mitochondrial data revealed clearly incongruent phylogenetic histories among different geographical populations as well as major DTUs. In parallel, we exploited read depth data, generated by Illumina sequencing of the maxicircle genome from the TcI reference strain Sylvio X10/1, to provide the first evidence of mitochondrial heteroplasmy (heterogeneous mitochondrial genomes in an individual cell) in *T. cruzi*.

**Conclusions/Significance:**

mtMLST provides a powerful approach to genotyping at the sub-DTU level. This strategy will facilitate attempts to resolve phenotypic variation in *T. cruzi* and to address epidemiologically important hypotheses in conjunction with intensive spatio-temporal sampling. The observations of both general and specific incidences of nuclear-mitochondrial phylogenetic incongruence indicate that genetic recombination is geographically widespread and continues to influence the natural population structure of TcI, a conclusion which challenges the traditional paradigm of clonality in *T. cruzi*.

## Author Summary

Chagas disease, caused by the protozoan parasite *Trypanosoma cruzi*, is an important public health problem in Latin America. While molecular techniques can differentiate the major *T. cruzi* genetic lineages, few have sufficient resolution to describe diversity among closely related strains. The online availability of three mitochondrial genomes allowed us to design a multilocus sequence typing (mtMLST) scheme to exploit these rapidly evolving markers. We compared mtMLST with current nuclear typing tools using isolates belonging to the oldest and most widely occurring lineage TcI. *T. cruzi* is generally believed to reproduce clonally. However, in this study, distinct branching patterns between mitochondrial and nuclear phylogenetic trees revealed multiple incidences of genetic exchange within different geographical populations and major lineages. We also examined Illumina sequencing data from the TcI genome strain which revealed multiple different mitochondrial genomes within an individual parasite (heteroplasmy) that were, however, not sufficiently divergent to represent a major source of typing error. We strongly recommend this combined nuclear and mitochondrial genotyping methodology to reveal cryptic diversity and genetic exchange in *T. cruzi*. The level of resolution that this mtMLST provides should greatly assist attempts to elucidate the complex interactions between parasite genotype, clinical outcome and disease distribution.

## Introduction

Mitochondrial genes are among the most popular markers for the reconstruction of evolutionary ancestries and resolution of phylogeographic relationships [1]. Their pervasive use in population genetics can be attributed to several intrinsic characteristics, notably, their high copy number, small size (∼15–20 kb) and faster mutation rate (compared with nuclear DNA). In addition, their widespread application is founded on the assumptions that mitochondrial genomes are homoplasmic, uniparentally inherited and lack homologous recombination [2]. However, with technological advances affording increased sensitivity and greater sample throughput, a growing number of reports of heteroplasmy (heterogeneous mitochondrial genomes in an individual cell), introgression and inter-molecular recombination are challenging what was previously regarded as a strict set of rules for eukaryotic mitochondrial inheritance.

Chagas disease remains the most important parasitic infection in Latin America, where an estimated 10–12 million individuals are infected, with a further 80 million at risk [3]. The aetiological agent, *Trypanosoma cruzi*, displays remarkable genetic diversity and is currently recognized as a complex of six lineages or discrete typing units (DTUs), each broadly associated with disparate ecologies and geographical distributions [4]. *T. cruzi* infection is life-long and can lead to debilitation and death by irreversible cardiac and/or gastrointestinal complications [5]. It has been suggested that the geographical heterogeneity in Chagas disease pathology is related to the genetic variation among *T. cruzi* DTUs [6], [7]. However, the relationship between parasite genotype and clinical outcome remains enigmatic. DTU nomenclature has recently been revised by international consensus to reflect the current understanding of *T. cruzi* genetic diversity [8]. Several evolutionary scenarios have been proposed to account for the emergence of two hybrid lineages (TcV and TcVI) and their parental progenitors (TcII and TcIII). However, the number of ancestral nuclear clades (two or three) remains controversial [9], [10].

TcI is the most abundant and widely dispersed of all *T. cruzi* lineages, with an ancient parental origin estimated at ∼0.5–0.9 MYA [11]. The distribution of domestic TcI, propagated by domiciliated triatomine vector species, principally extends from the Amazon Basin northwards, where it is implicated as the main cause of Chagas disease in endemic areas such as Venezuela and Colombia [12], [13]. TcI is also ubiquitous in sylvatic transmission cycles throughout South America and extends into North and Central America [14], [15]. Recent advances in new high resolution genotyping techniques have seen a resurgence of interest in unravelling TcI intra-lineage diversity. In Colombia, sequencing of the mini-exon spliced leader intergenic region (SL-IR) has subdivided TcI isolates from domestic and sylvatic transmission cycles, irrespective of geographical origin [16]–[18]. Other studies have demonstrated geographical clustering of TcI strains and an ecological association between specific genotypes and *Didelphis* hosts [19]. Higher resolution studies exploiting multiple microsatellite markers (MLMT) also report limited gene flow between sylvatic and domestic transmission cycles manifesting as genetic diversity between TcI isolates from sympatric sites [20], [21]. In addition, unexpectedly high levels of homozygosity in multiple clones from single hosts may be indicative of recombination between similar genotypes (inbreeding) or recurrent, genome wide, and dispersed gene conversion [20], [22]. The frequency and mechanism of natural intra-TcI genetic exchange are thus unknown, largely due to inappropriate or inadequate sampling. Evidence for such recombination is increasing and has already been documented among strains isolated from sylvatic *Didelphis* and *Rhodnius* in the Amazon Basin [23] and within a domestic/peridomestic TcI population in Ecuador [21]. Furthermore, the generation of intra-lineage TcI hybrids *in vitro* indicates that this ancestral lineage has an extant capacity for genetic exchange [24].

In kinetoplastids, the mitochondrial genome is represented by 20–50 maxicircles (20–40 kb) which, together with thousands of minicircles (0.5–10 kb), form a catenated network or kinetoplast (kDNA), comprising 20–25% of total cellular DNA [25]. Maxicircles are the functional equivalent of eukaryotic mitochondrial DNA, encoding genes for mitochondrial rRNAs and hydrophobic proteins involved in energy transduction by oxidative phosphorylation [26]. Previously, phylogenetic analyses of *T. cruzi* maxicircle fragments classified isolates into three mitochondrial clades A (TcI), B (TcIII, TcIV, TcV and TcVI) and C (TcII) [10], [27]. To date, maxicircle typing has been principally used to examine *T. cruzi* inter-lineage diversity, with sequencing efforts reliant on a limited number of genes [28] and often in the absence of any comparative nuclear targets [29], [30]. However, the inherent features of mitochondrial markers argue for their inclusion as principal but not solitary components of phylogenetic studies. Indeed, the caveats highlighted by other eukaryotes are especially pertinent with respect to *T. cruzi*. Mitochondrial introgression has been reported in North America where identical maxicircles circulate in sympatric TcI and TcIV from sylvatic reservoirs [27] and in South America where maxicircle haplotypes are shared between TcIII and TcIV strains with highly divergent nuclear genomes [11]. However, this phenomenon has not been described among South American TcI isolates. In addition, mitochondrial heteroplasmy, a possible confounder of phylogenetic studies, has not been examined in the coding region of the *T. cruzi* maxicircle but is not unexpected considering the presence of up to fifty maxicircle copies within an individual parasite.

The potential for mitochondrial DNA to reveal diversity hidden at the sub-DTU level in *T. cruzi* has been largely overlooked. To address this deficit, we first employed a whole genome approach to investigate the existence of maxicircle heteroplasmy and to resolve its role as a source of genotyping error. Secondly, we exploited the online availability of three complete *T. cruzi* maxicircle genomes [31], [32] to develop a high resolution mitochondrial multilocus typing scheme (mtMLST) in order to describe TcI intra-lineage diversity. Lastly, we investigated the extent of incongruence between mitochondrial and nuclear loci (SL-IR, *GPI* and 25 short tandem repeat (STR) loci) to detect incidences of genetic exchange.

## Materials and Methods

### Illumina Sequencing of the Sylvio X10/1 Maxicircle Genome

The maxicircle genome from Sylvio X10/1 (TcI) was sequenced at 183X coverage using Illumina HiSeq 2000 technology as part of the Sylvio X10/1 Whole Genome Shotgun project [33]. A total of 66,882 reads were generated which covered the maxicircle coding region (15,185 bp). The consensus maxicircle genome sequence was derived from the predominant nucleotide present across multiple read alignments at each position. However, this criterion masks minor maxicircle haplotypes (evidence of heteroplasmy) by disregarding low abundance single nucleotide polymorphisms (SNPs). To assess the presence/absence of true minor SNPs, all 66,882 reads were re-aligned to the Sylvio X10/1 maxicircle genome using the alignment software SAMtools [34] and SNPs were called using the SAMtools mpileup commands. A SNP was defined as a nucleotide variant present in at least 5 independent reads (with parameters: 20X coverage; and mapping quality, 30). The final alignment was manually inspected using Tablet [35]. In parallel, ten maxicircle gene fragments, described below, were amplified by PCR and Sanger sequenced from Sylvio X10/1.

### Strains

A panel of 32 TcI isolates was assembled for analysis (Table 1). Parasites (epimastigotes) were cultured at 28°C in RPMI-1640 liquid medium supplemented with 0.5% (w/v) tryptone, 20 mM HEPES buffer pH 7.2, 30 mM haemin, 10% (v/v) heat-inactivated fetal calf serum, 2 mM sodium glutamate, 2 mM sodium pyruvate and 25 µg/ml gentamycin (Sigma, UK) [23]. Genomic DNA was extracted using the Gentra PureGene Tissue Kit (Qiagen, UK), according to the manufacturer's protocol. Isolates were previously characterized to DTU level using a triple-marker assay [36] and classified into seven genetic populations by microsatellite profiling [20]: North and Central America (AM_North/Cen_), Venezuelan sylvatic (VEN_silv_), North-Eastern Brazil (BRAZ_North-East_), Northern Bolivia (BOL_North_), Northern Argentina (ARG_North_), Bolivian and Chilean Andes (ANDES_Bol/Chile_) and Venezuelan domestic (VEN_dom_). Genotypes for additional TcI–TcVI strains were included for comparison in selected analyses as indicated (Tables S1 and S2).

**Table 1:** Panel of *T. cruzi* isolates assembled for analysis.

Strain Code*	Strain	Date of isolation	Host/Vector	Locality	Latitude	Longitude	Population
9209	92090802P cl1	1992	*Didelphis marsupialis*	Georgia, USA	32.43	−83.31	*AM*_North/Cen_
9307	93070103P cl1	1993	*Didelphis marsupialis*	Georgia, USA	32.43	−83.31	*AM*_North/Cen_
DAVIS	DAVIS 9.90 cl1	1983	*Triatoma dimidiata*	Tegucigalpa, Honduras	14.08	−87.2	*AM*_North/Cen_
OPOS	USAOPOSSUM cl2	Unknown	*Didelphis marsupialis*	Louisiana, USA	30.5	−91	*AM*_North/Cen_
ARMA	USAARMA cl3	Unknown	*Dasypus novemcinctus*	Louisiana, USA	30.5	−91	*AM*_North/Cen_
COT38	COTMA38	13.10.04	*Akodon boliviensis*	Cotopachi, Bolivia	−17.43	−66.27	*ANDES*_Bol/Chile_
P234	P234	1985	*Homo sapiens*	Cochabamba, Bolivia	−17.38	−66.16	*ANDES*_Bol/Chile_
P238	P238	1985	*Homo sapiens*	Cochabamba, Bolivia	−17.38	−66.16	*ANDES*_Bol/Chile_
P268	P268	1987	*Homo sapiens*	Cochabamba, Bolivia	−17.38	−66.16	*ANDES*_Bol/Chile_
PAL23	PALDAV2∧3	23.03.01	*Triatoma infestans*	Chaco, Argentina	−27.133	−61.46	*ARG*_North_
PAL4	PALDA4	23.03.01	*Didelphis albiventris*	Chaco, Argentina	−27.133	−61.46	*ARG*_North_
PAL5	PALDA5	23.03.01	*Didelphis albiventris*	Chaco, Argentina	−27.133	−61.46	*ARG*_North_
PAL20	PALDA20	23.03.01	*Didelphis albiventris*	Chaco, Argentina	−27.133	−61.46	*ARG*_North_
PAL21	PALDA21	23.03.01	*Didelphis albiventris*	Chaco, Argentina	−27.133	−61.46	*ARG*_North_
SJ34	SJM34	07.09.04	*Didelphis marsupialis*	Beni, Bolivia	−14.81	−64.6	*BOL*_North_
SJ41	SJM41	09.09.04	*Philander opossum*	Beni, Bolivia	−14.81	−64.6	*BOL*_North_
SJ37	SJM37	09.09.04	*Didelphis marsupialis*	Beni, Bolivia	−14.81	−64.6	*BOL*_North_
SJ12	SJMC12	13.09.04	*Philander opossum*	Beni, Bolivia	−14.81	−64.6	*BOL*_North_
SJ22	SJM22 cl1	06.09.04	*Didelphis marsupialis*	Beni, Bolivia	−14.81	−64.6	*BOL*_North_
SJ39	SJM39 cl3	09.9.04	*Didelphis marsupialis*	Beni, Bolivia	−14.81	−64.6	*BOL*_North_
M13	M13	12.06.04	*Didelphis marsupialis*	Barinas, Venezuela	7.5	−71.23	*VEN*_silv_
M16	M16 cl4	13.06.04	*Didelphis marsupialis*	Barinas, Venezuela	7.5	−71.23	*VEN*_silv_
M18	M18	13.06.04	*Didelphis marsupialis*	Barinas, Venezuela	7.5	−71.23	*VEN*_silv_
M7	M7	14.05.04	*Didelphis marsupialis*	Barinas, Venezuela	7.5	−71.23	*VEN*_silv_
XE51	XE5167 cl1	14.09.99	*Didelphis marsupialis*	Para, Brazil	−1.71	−48.88	*BRAZ*_North-East_
IM48	IM4810	23.04.02	*Didelphis marsupialis*	Manaus, Brazil	−3.07	−60.16	*BRAZ*_North-East_
XE29	XE2929	10.08.88	*Didelphis marsupialis*	Pará, Brazil	−5.83	−48.03	*BRAZ*_North-East_
B2085	B2085	03.01.91	*Didelphis marsupialis*	Belem, Brazil	−1.36	−48.36	*BRAZ*_North-East_
1180	11804	2003	*Homo sapiens*	Portuguesa, Venezuela	9.01	−69.29	*VEN*_dom_
1171	11713	2003	*Homo sapiens*	Lara, Venezuela	10.04	−69.32	*VEN*_dom_
9354	9354	1999	*Homo sapiens*	Sucre, Venezuela	10.46	−63.61	*VEN*_dom_
1154	11541	2003	*Homo sapiens*	Merida, Venezuela	8.59	−71.23	*VEN*_dom_

*: Strain code corresponds to labels on Figure 3 and descriptions in text.

### Maxicircle Genes (mtMLST)

**Figure 1:**
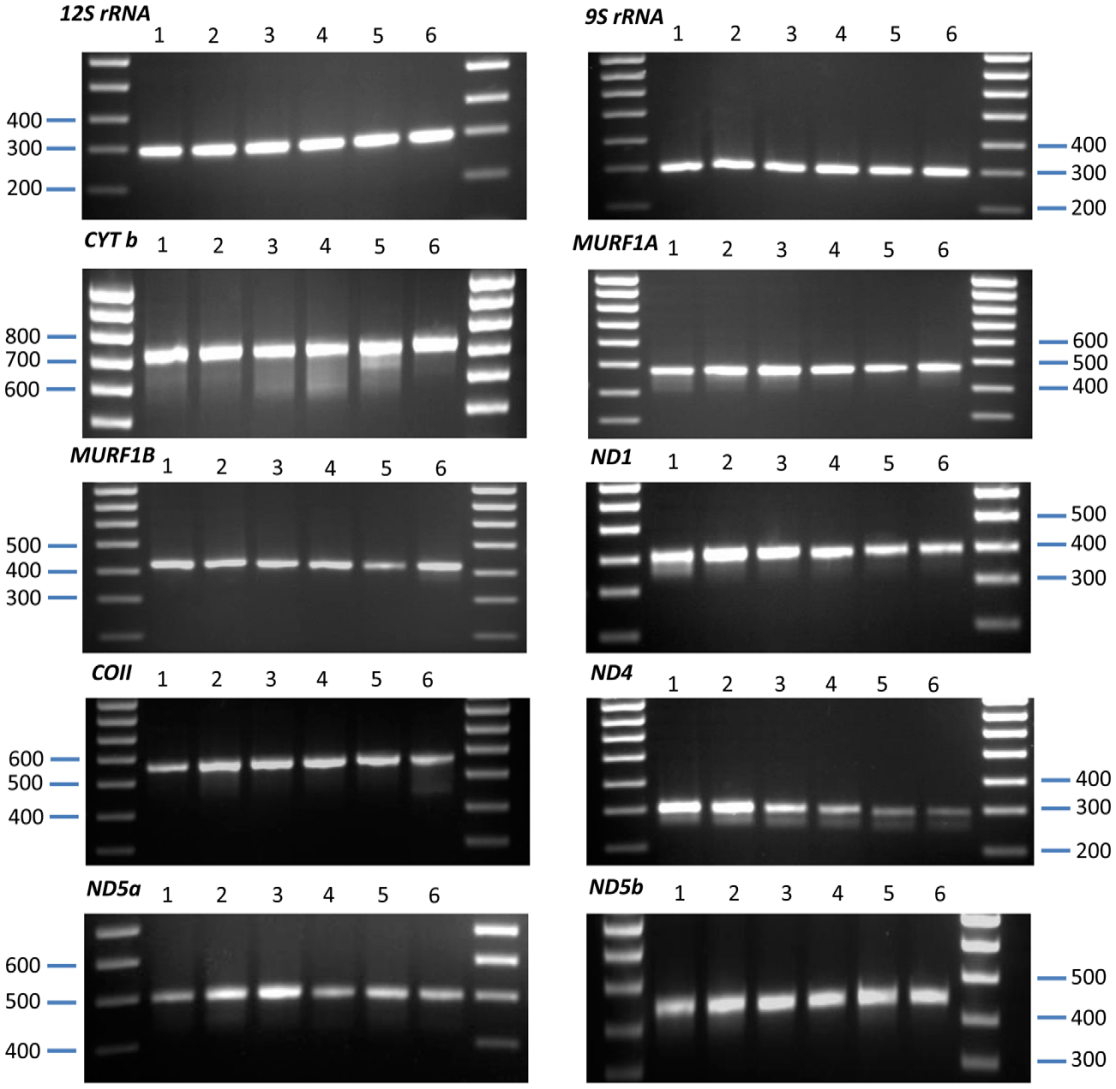
PCR products from ten maxicircle gene fragments amplified across the six *T. cruzi* DTUs. Amplification products were visualized on 1.5% agarose gels stained with ethidium bromide. Molecular weight marker is Hyperladder IV (Bioline, UK). For all gels: lane 1 - Sylvio X10/1 (TcI), lane 2 - Esm cl3 (TcII), lane 3 - M5631 cl5 (TcIII), lane 4 - CanIII cl1 (TcIV), lane 5 - Sc43 cl1 (TcV), and lane 6 - CL Brener (TcVI). Robust amplification was observed for the ten maxicircle gene fragments across reference isolates belonging to the six DTUs.

Ten maxicircle gene fragments were amplified: *ND4* (NADH dehydrogenase subunit 4), *ND1* (NADH dehydrogenase subunit 1), *COII* (cytochrome *c* oxidase subunit II), *MURF1* (Maxicircle unidentified reading frame 1, two fragments), *CYT b* (cytochrome *b*), *12S rRNA*, *9S rRNA*, and *ND5* (NADH dehydrogenase subunit 5, two fragments) coding regions. Degenerate primers were designed in primaclade [37] using complete maxicircle reference sequences from CL Brener (TcVI), Sylvio X10/1 (TcI), and Esm cl3 (TcII) available online at www.tritrypdb.org [38]. Primer sequences and annealing temperatures for PCR amplifications are given in Table 2. Robust amplification was first confirmed across a reference panel of all six *T. cruzi* DTUs (see Table S1 and Figure 1).

**Table 2:** *T. cruzi* maxicircle gene fragments and primer details.

Gene Fragment	Genome Position^a^	Primer Name	Primer Sequence (5′→3′)	Annealing Temp. (°C)	Amplicon Size (bp)^b^	Sequence Start 5′	Sequence Start 3′	Sequenced Fragment (bp)^c^	Accession Numbers
*12S rRNA*	639–901	*12S* Fwd	GTTTATTAAATGCGTTTGTCTAAGAA (26)	50	299	GTCTAAGA	TACGTATT	263	JQ581254–JQ581292
		*12S* Rvs	GCCCCAATCAAACATACAA (19)						
*9S rRNA*	1077–1309	*9S* Fwd	TGCAATTCGTTAGTTGGGTTA (21)	50	302	TAAAATCG	TATTATTA	233	JQ581215–JQ581253
		*9S* Rvs	TCCACACCCATTAAATAGCACT (22)						
*CYT b*	4126–4733	*Sp18* Fwd	GACAGGATTGAGAAGCGAGAGAG (23)	50	717	TTTGTYTT	TAATAYCA	608	JQ581332–JQ581370
		*Sp18* Rvs	CAAACCTATCACAAAAAGCATCTG (24)						
*Murf1*a	6011–6393	*Murf1*a Fwd	AAGGCRATGGGRATAGWRCCTATAC (25)	50	482	ACTAAGYA	ACTTTYTA	383	JQ581403–JQ581441
		*Murf1*a Rvs	TGGAACAATTRTATATCAGATTRGGA (26)						
*Murf1*b	6528–6900	*Murf1*b Fwd	ACMCCCATCCATTCTTCR (18)	50	423	CAAAAATT	GGATTTAT	373	JQ581442–JQ581480
		*Murf1*b Rvs	CCTTTGATYTATTGTGATTAACRKT (25)						
*ND1*	7643–8011	*ND1* Fwd	GCACTTTCTGAAATAATCGAAAA (23)	50	400	TCGAAAAA	TTGTTAGC	369	JQ581059–JQ581097
		*ND1* Rvs	TTAATCTTATCAGGATTTGTTAGCC (25)						
*COII*	8194–8610	*COII* Fwd	GTTATTATCTTTTGTTTGTTTTGTGTG (27)	50	560	CTTTCTAC	ACCTRCCY	417	JQ581293–JQ581331
		*COII* Rvs	AACAATTGGCATAAATCCATGT (22)						
*ND4*	12153–12392	*ND4* Fwd	TTTTTGAAAGTCTATTTTTCCCA (23)	50	302	AATTTTAA	CGGTYRTC	240	JQ581098–JQ581136
		*ND4* Rvs	CTTCAACATGCATTTCCGGTT (21)						
*ND5*a	13829–14250	*ND5*a Fwd	TATGRYTAACYTTTTCATGYTCRG (24)	50	503	GTACATAY	TYTTYGTA	422	JQ581137–JQ581175
		*ND5*a Rvs	GTCCTTCCATYGCATCYGG (19)						
*ND5*b	14274–14640	*ND5*b Fwd	ARAGTACACAGTTTGGRYTRCAYA (24)	50	444	TGATTRCC	GYARACCA	367	JQ581176–JQ581214
		*ND5*b Rvs	CTTGCYAARATACAACCACAA (21)						

a Genome position according to the TcI Sylvio X10/1 reference maxicircle genome [33].

b Amplicon size according to TcI Sylvio X10/1. Indels in other strains may cause size variation.

c Sequence length according to TcI Sylvio X10/1. Indels in other strains may cause length variation.

Amplifications for all targets were achieved in a final volume of 20 µl containing: 1× NH_4_ reaction buffer, 1.5 mM MgCl_2_ (Bioline, UK), 0.2 mM dNTPs (New England Biolabs, UK), 10 pmol of each primer, 1 U *Taq* polymerase (Bioline, UK) and 10–100 ng of genomic DNA. PCR reactions were performed with an initial denaturation step of 3 minutes at 94°C, followed by 30 amplification cycles (94°C for 30 seconds, 50°C for 30 seconds, 72°C for 30 seconds) and a final elongation step at 72°C for ten minutes. PCR products were purified using QIAquick PCR extraction kits (Qiagen, UK) according to the manufacturer's protocol.

### Nuclear Genes

The mini-exon spliced leader intergenic region (SL-IR) and glucose-6-phosphate isomerase (*GPI*) were amplified as previously described by Souto *et al.* (1996) [39] and Lewis *et al.* (2009) [36], respectively. PCR products were visualized in 1.5% agarose gels and if necessary purified using QIAquick PCR and gel extraction kits (Qiagen, UK) to remove non-specific products. Bi-directional sequencing was performed for both nuclear and maxicircle targets using the BigDye® Terminator v3.1 Cycle Sequencing Kit (Applied Biosystems, UK) according to the manufacturer's protocol. Maxicircle PCR products were sequenced using the relevant PCR primers described in Table 2. Nuclear amplicons were sequenced using their respective PCR primers. When ambiguous sequences were obtained, PCR products were cloned into the pGEM® - T Easy Vector System I (Promega, UK), according to the manufacturer's instructions, and transformed into XL1-Blue *E. coli* (Agilent Technologies, UK), prior to colony PCR and re-sequencing. For strains that produced incongruent nuclear and maxicircle phylogenetic signals, PCR and sequencing reactions were replicated twice using DNA derived from two independent genomic DNA extractions.

### Microsatellite Loci

Data from 25 previously described microsatellite loci [20], distributed among ten chromosomes [40], were included for analysis. Loci were selected from a wider panel of 48 microsatellite loci based on their level of TcI intra-lineage resolution. In addition, these 25 microsatellite loci were amplified across eight new unpublished biological clones (M16 cl4, SJM22 cl1, SJM39 cl3, USAARMA cl3, USAOPOSSUM cl2, 92090802P cl1, 93070103P cl1 and DAVIS 9.90 cl1). Primers and binding sites are listed in Table S3. The following reaction conditions were implemented across all loci: a denaturation step of 4 minutes at 95°C, then 30 amplification cycles (95°C for 20 seconds, 57°C for 20 seconds, 72°C for 20 seconds) and a final elongation step at 72°C for 20 minutes. Amplifications were achieved in a final volume of 10 µl containing: 1× ThermoPol Reaction Buffer (New England Biolabs, UK), 4 mM MgCl_2_, 34 µM dNTPs, 0.75 pmol of each primer, 1 U *Taq* polymerase (New England Biolabs, UK) and 1 ng of genomic DNA. Five fluorescent dyes were used to label the forward primers: 6-FAM and TET (Proligo, Germany) and NED, PET and VIC (Applied Biosystems, UK). Allele sizes were determined using an automated capillary sequencer (AB3730, Applied Biosystems, UK), in conjunction with a fluorescently tagged size standard, and were manually checked for errors. All isolates were typed “blind” to control for user bias.

### Phylogenetic Analysis of Nuclear Loci

Pair-wise distances (*D*_AS_) between microsatellite genotypes for individual samples were calculated in MICROSAT v1.5d [41] under the infinite-alleles model (IAM). To accommodate multi-allelic genotypes (≥3 alleles per locus), a script was written in Microsoft Visual Basic to generate random multiple diploid re-samplings of each multilocus profile (software available on request). A final pair-wise distance matrix was derived from the mean of each re-sampled dataset and used to construct a Neighbour-Joining phylogenetic tree in PHYLIP v3.67 [42]. Majority rule consensus analysis of 10,000 bootstrap trees was performed in PHYLIP v3.67 by combining 100 bootstraps created in MICROSAT v1.5d, each drawn from 100 respective randomly re-sampled datasets.

Nucleotide sequences were assembled manually in BioEdit v7.0.9.0 sequence alignment editor software (Ibis Biosciences, USA) [43] and unambiguous consensus sequences were produced for each isolate. Heterozygous SNPs were identified by the presence of two coincident peaks at the same locus (‘split peaks’), verified in forward and reverse sequences and scored according to the one-letter nomenclature for nucleotides from the International Union of Pure and Applied Chemistry (IUPAC). For both nuclear genes (SL-IR and *GPI*), edited sequences were used to generate Neighbour-Joining trees based on the Kimura-2 parameter model in MEGA v5 [44]. Bootstrap support for clade topologies was estimated following the generation of 1000 pseudo-replicate datasets. Once both trees were visualized independently to confirm congruent topologies, nuclear SNPs were re-coded numerically and concatenated with microsatellite data (see Dataset S1). *D*_AS_ values were calculated for the concatenated dataset as described above and used to generate a single Neighbour-Joining phylogenetic tree encompassing all nuclear genetic diversity. Nucleotide sequences for *GPI* and the SL-IR are available from GenBank under the accession numbers JQ581371–JQ581402 and JQ581481–JQ581512, respectively.

### Phylogenetic Analysis of Maxicircle Genes

Sequence data were assembled manually as described for nuclear loci. For each isolate, maxicircle sequences were concatenated according to their structural arrangement (*12S rRNA*, *9S rRNA*, *CYT b*, *MURF1*, *ND1*, *COII*, *ND4* and *ND5*) and in the correct coding direction (alignment available on request). Nucleotide sequences for all ten gene fragments are available from GenBank under the accession numbers listed in Table 2. Phylogenies were inferred using Maximum-Likelihood (ML) implemented in PhyML (4 substitution rate categories) [45]. The best-fit model of nucleotide substitution was selected from 88 models and its significance evaluated according to the Akaike Information Criterion (AIC) in jMODELTEST 1.0. [46]. The best model selected for this dataset was GTR+I+G. Bootstrap support for clade topologies was estimated following the generation of 1000 pseudo-replicate datasets. Bayesian phylogenetic analysis was performed using MrBAYES v3.1 [47] (settings according to jMODELTEST 1.0). Five independent analyses were run using a random starting tree with three heated chains and one cold chain over 10 million generations with sampling every 10 simulations (25% burn-in). Shimodaira-Hasegawa likelihood tests (SH tests) [48] were implemented in PAML v.4 [49] to statistically evaluate incongruencies between alternative tree topologies derived from the mitochondrial and nuclear data.

## Results

### Maxicircle Heteroplasmy

**Figure 2:**
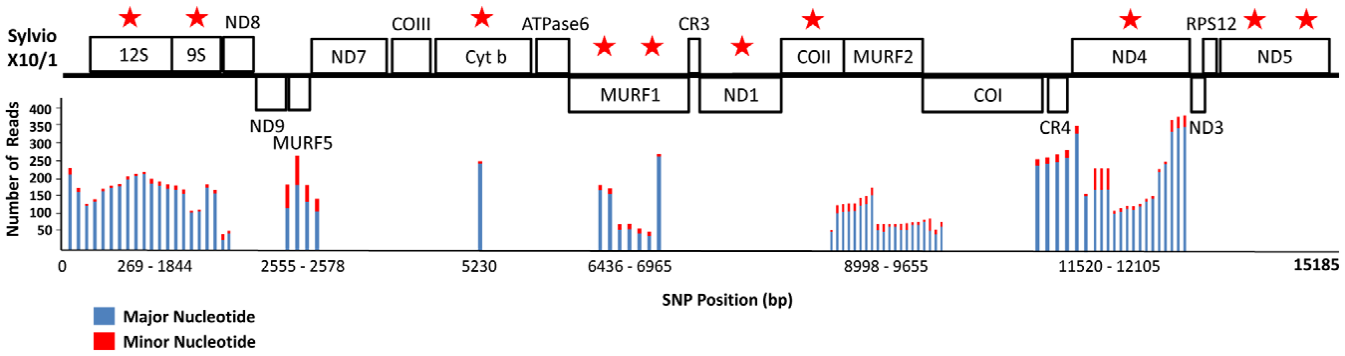
Distribution of seventy-four heteroplasmic sites across the 15, 185 bp Sylvio X10/1 maxicircle genome (schematic shows linearized maxicircle). 66,882 sequencing reads covering the Sylvio X10/1 maxicircle were generated using Illumina HiSeq 2000 technology as part of the Sylvio X10/1 Whole Genome Shotgun project. Multiple reads were re-aligned to the maxicircle genome and SNPs were identified if a nucleotide variant was present in at least five independent reads. Bars represent the abundance of major (reference nucleotide) and minor bases among multiple reads at each position. All SNPs are bi-variable. At some overlapping positions, different contiguous SNPs are distributed among separate sequencing reads. These observations suggest the occurrence of at least two additional maxicircle genomes at a ∼10-fold lower abundance compared to the consensus genome. Red stars denote gene fragments used in the mtMLST scheme.

Across the 15,185 bp of the Sylvio X10/1 maxicircle coding region a total of 74 SNPs were identified among eight genes (*12S rRNA*, *9S rRNA*, *MURF5*, *CYT b*, *MURF1*, *MURF2*, *CR4* and *ND4*) and three intergenic regions (between *12S rRNA* and *9S rRNA*, between *9S rRNA* and *ND8* and between *CR4* and *ND4*, respectively) (Figure 2 and Table S4). Average read depth for each SNP site was 163. At heterozygous sites, the minor nucleotide was present among an average of 12.2% (±9.1%) of sequence reads. In each gene, SNPs were clustered often <5 bp apart in pairs and triplets. The most common mutations were transversions from A→T (14/74), T→A (10/74), T→G (7/74) and G→T (6/74) and transitions from A→G (13/74). SNPs were bi-variable at all sites. The presence of different contiguous SNPs distributed across separate sequencing reads at overlapping positions suggests the occurrence of at least two minor maxicircle templates within the same sample. However, the short average length of Illumina reads (∼100 bp) prohibits the full reconstruction of minor maxicircle sequence types. No evidence of heterozygosity was observed in any of the ten maxicircle Sanger sequences (from the mtMLST scheme) that covered the corresponding areas of heteroplasmy identified in Sylvio X10/1, which is consistent with the low sensitivity of this method.

### Maxicircle Genes (mtMLST)

Degenerate primers were designed by reference to complete TcI, TcII and TcVI maxicircle genomes. Ten gene fragments from eight maxicircle coding regions were selected in order to sample genetic diversity present across the whole *T. cruzi* maxicircle. For two genes (*MURF1* and *ND5*) two fragments were selected from each coding region to examine intra-gene variation. Reliable PCR amplification of all ten maxicircle fragments was first confirmed using a panel of *T. cruzi* reference strains from each DTU (see Figure 1).

The maxicircle gene targets were then sequenced across the TcI panel (Table 1) and seven additional TcIII/TcIV strains (Table S2). Relatively uniform substitution rates were observed among all genes (gamma shape parameter α = 0.8121, based on the GTR+I+G model). For each TcI isolate, gene fragments were concatenated according to their structural position and assembled into a 3686 bp alignment. Twenty-two unique haplotypes were identified from a total of 355 variable sites (∼9.6% sequence diversity). No evidence of heterozygosity (‘split peaks’) was observed.

**Figure 3:**
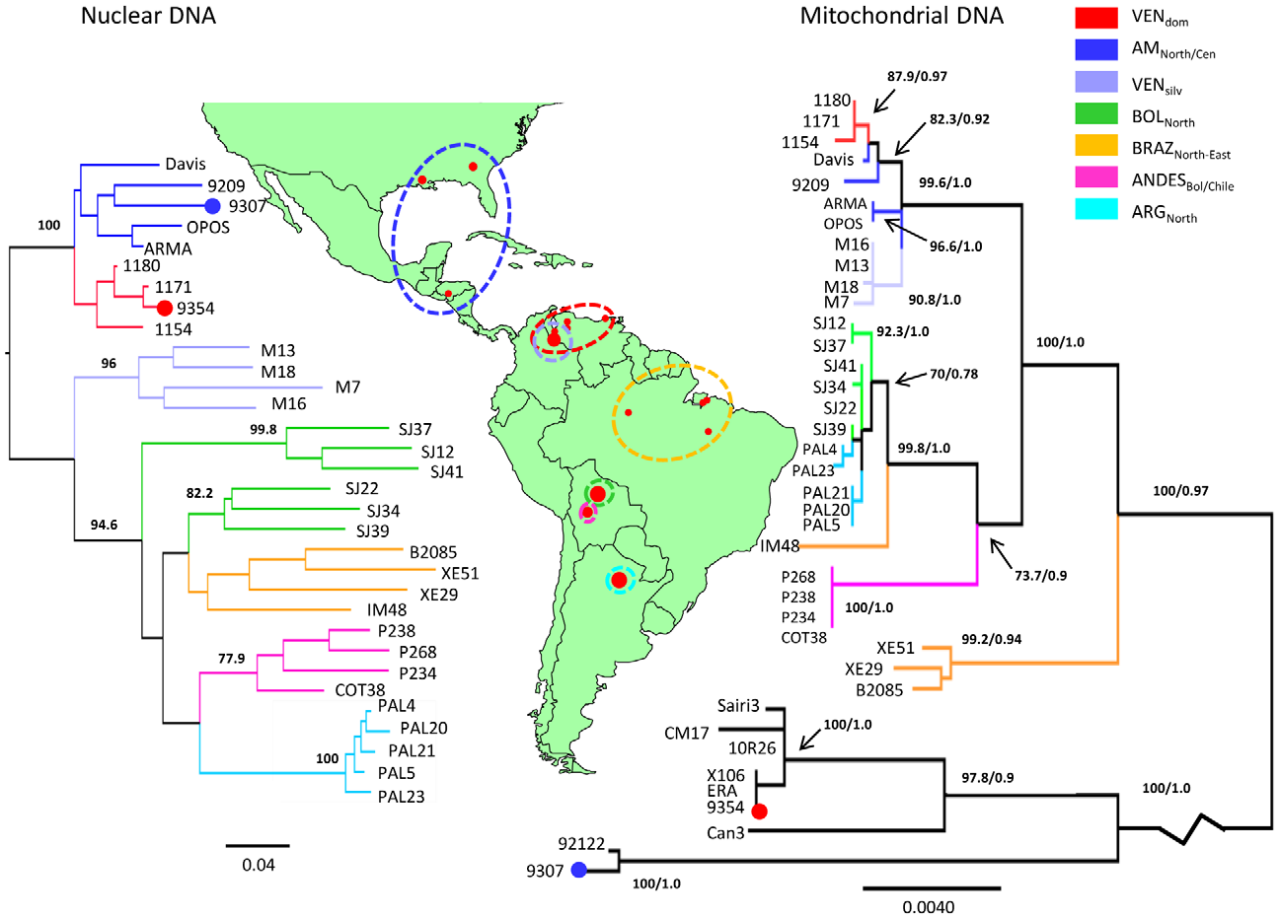
Unrooted Neighbour-Joining tree based on D_AS_ values from nuclear loci (left) and Maximum-Likelihood tree from concatenated maxicircle sequences (right) showing TcI population structure across the Americas. A panel of 32 TcI isolates from seven nuclear populations was assembled for analysis. Origin of individual strains is shown on the map by small red circles. Large red circles correspond to multiple samples, isolated from the same geographical area. Branch colours indicate strain population. The nuclear tree was constructed from concatenated polymorphisms present within the SL-IR, *GPI* and 25 microsatellite loci. *D*_AS_ values were calculated as the mean across 1000 random diploid re-samplings of the dataset and those greater than 70% are shown on major clades. A Maximum-Likelihood topology was assembled from concatenated maxicircle sequences. Branches show equivalent bootstraps and posterior probabilities from consensus Maximum-Likelihood (1000 replicates) and Bayesian topologies, respectively. The maxicircle topology is rooted against additional outgroup strains from TcIII and TcIV. The blue and red circles on branches represent inter-lineage introgression events. The blue circle indicates that the maxicircle in a sylvatic TcI isolate from AM_North/Cen_ is most closely related to the maxicircle found in TcIV samples from the same area. The red circle shows that the maxicircle haplotype in a human VEN_dom_ strain is the same as those in TcIII and TcIV isolates from neighbouring areas of Venezuela, Bolivia and Colombia. Divergent maxicircle haplotypes at the intra-DTU level are also observed in BRAZ_North-East_ (IM48) and AM_North/Cen_ (ARMA and OPOS). Another incidence of nuclear-mitochondrial incongruence is demonstrated by the paraphyletic grouping of ARG_North_ among a subset of BOL_North_ isolates in the maxicircle tree, compared to its monophyletic placement in the nuclear phylogeny.

Maximum-Likelihood (Figure 3, right) and Bayesian phylogenies were both constructed from the concatenated maxicircle data. No statistically-supported incongruence was observed between the two topologies (Bayesian tree *L* = −6770.21, ML tree *L* = −6768.85, *P* = 0.428). The presence of at least three incongruent haplotypes (see below) precludes the accurate clustering of their respective populations (AM_North/Cen_, VEN_dom_ and BRAZ_North-East_). However, phylogenetic analysis does resolve two well-supported clades corresponding to VEN_silv_ and ANDES_Bol/Chile_ (90.8%/1.0 and 100%/1.0, respectively). Once the two TcIV-type maxicircles were excluded from analysis, the mtMLST was re-evaluated with respect to intra-TcI discriminatory power. One hundred SNPs were identified among 3681 bp (∼2.7% sequence diversity), corresponding to twenty maxicircle haplotypes. Both Bayesian and Maximum-Likelihood topologies were congruent with those constructed previously for the entire TcI isolate panel.

### Nuclear Loci

The resolutive power of the mtMLST scheme was evaluated by comparison to current markers used to investigate TcI intra-DTU nuclear diversity, specifically, a housekeeping gene (*GPI*), a non-coding multi-copy intergenic region (SL-IR) and a MLMT panel of 25 loci. Sequences for *GPI* were obtained for 32 *T. cruzi* isolates (Table 1) and assembled into a gap-free alignment of 921 nucleotides. Of the 921 bp, a total of 911 invariable sites and 10 polymorphic sites were identified (∼1.1% sequence diversity). A 350 bp alignment corresponding to the SL-IR was generated for the same panel of samples. Strains from two populations (5/6 BOL_North_ and 4/4 ANDES_Bol/Chile_) presented sequences with multiple ambiguous base calls due to the presence of a GT_n_ microsatellite at positions 14–24. For these nine isolates, haplotypes were determined by sequencing four cloned PCR products to derive a consensus sequence. In the 350 bp alignment, 323 conserved sites and 36 polymorphic sites were observed (∼10.3% sequence diversity). All samples were also typed at 25 polymorphic microsatellite loci yielding a total of 1612 alleles. The majority of strains presented one or two alleles at each locus. Multiple alleles (≥3) were observed at a small proportion of loci (1.5%).

Individual Neighbour-Joining trees were re-constructed for *GPI*, SL-IR and the MLMT data. No well-supported sub-DTU level clades were recovered using *GPI* sequences. The SL-IR phylogeny resolved two populations (VEN_silv_ and ARG_North_) with strong statistical support (85% and 99%, respectively; data not shown). Three major clades were identified by MLMT (VEN_dom_, ARG_North_ and ANDES_Bol/Chile_) with good bootstrap support (72.6%, 99.3% and 98.4%, respectively; data not shown). There was no bootstrap-supported incongruence between the three nuclear tree topologies. This justified their concatenation and these data were re-coded and analyzed in a single distance-based phylogeny (independent of mutation rate heterogeneity) (Figure 3, left and Dataset S1). The concatenated nuclear tree recovered three well supported clades corresponding to TcI populations (VEN_silv_, ARG_North_ and ANDES_Bol/Chile_) (96%, 100% and 77.9%, respectively, Figure 3). Isolates belonging to the VEN_dom_ population remained grouped together but with a minor reduction in bootstrap values (64.8%), compared to the MLMT tree. In addition, the concatenated tree also subdivided BOL_North_ into two well defined sympatric clades each containing three isolates (99.8% and 82.2%). No nuclear targets (either individually or concatenated) were able to reliably identify AM_North/Cen_, or BRAZ_North-East_ as discrete clusters. However, AM_North/Cen_ was more closely related to VEN_dom_ than any other population by MLMT (90.2%), the SL-IR (99%) and the concatenated nuclear tree (100%).

### Nuclear-Mitochondrial Incongruence

Comparison of the mitochondrial and nuclear phylogenies revealed clear incongruence at multiple scales. The nuclear topology was a significantly worse model to fit the maxicircle data (nuclear tree *L* = −7008.72, mtMLST ML tree *L* = −6554.50, *P*<0.001). Three individual isolates had unambiguously different phylogenetic positions between the nuclear and mitochondrial datasets: 9307, 9354 and IM48 (Figure 3). The maxicircle sequences from 9307, a sylvatic TcI AM_North/Cen_ strain, and 9354, a human TcI strain from VEN_dom_, were divergent from all other TcI strains. Comparison with sequences from other DTUs indicates that the maxicircle from 9307 was most closely related to those found in TcIV samples from North America (92122) (100%/1.0) while 9354 shared its mitochondrial haplotype with TcIV and TcIII strains from neighbouring areas of Venezuela, Bolivia and Colombia (ERA, 10R26, X106, Sairi3 and CM17) (97.8%/0.9). IM48 from BRAZ_North-East_ also had a distinct maxicircle haplotype that formed a long branch separated from the other members of this population whereas for nuclear data all BRAZ_North-East_ isolates, including IM48, clearly grouped together.

To test whether inclusion of these isolates could explain the overall incongruence, the SH analysis was repeated for alternative nuclear vs. mitochondrial topologies with each of these strains excluded individually and then collectively. In all cases, statistically significant incongruence persisted (no 9307 *P* = 0.004, no 9354 *P* = 0.002, no IM48 *P*<0.001 and without all three *P* = 0.008). This indicated that mitochondrial introgression was generally pervasive in the TcI panel beyond these three isolates. For example, ARG_North_ samples, which formed a homogeneous monophyletic clade that was most closely related to ANDES_Bol/Chile_ by nuclear data, grouped paraphyletically amongst subsets of BOL_North_ strains in the maxicircle tree. In addition, BRAZ_North-East_ is grouped with one of the BOL_North_ clades in the nuclear tree, but receives a basally diverging position in the maxicircle phylogeny. In agreement with the nuclear data, AM_North/Cen_ was most closely related to VEN_dom_. However, two isolates from AM_North/Cen_ (ARMA and OPOS) displayed an unexpected level of maxicircle diversity and are grouped separately with strong bootstrap support (96.6%/1.0).

## Discussion

Elucidating the complex epidemiology, phylogeography and taxonomy of *T. cruzi* requires a clear understanding of the parasite's genetic diversity [4]. One objective of this study was to develop the first mitochondrial (maxicircle) multilocus sequence typing scheme (mtMLST) to investigate *T. cruzi* intra-lineage diversity and to critically assess its resolutive power compared to the current repertoire of phylogenetic markers.

The presence of intra-strain maxicircle diversity within Sylvio X10/1 is the first demonstration of heteroplasmy in the coding region of a *T. cruzi* maxicircle genome. Seventy-four variable sites were identified by read depth analysis of Illumina sequence data but undetected by conventional Sanger sequencing. These SNPs indicate the occurrence of at least two additional maxicircle genomes, present at a ∼10-fold lower abundance compared to the consensus published Sylvio X10 maxicircle genome [32]. Most heteroplasmic SNPs were linked. This may indicate an older most recent common ancestor (MRCA) between the major and minor maxicircles than that expected to have emerged in culture post-cloning. Thus these minor maxicircle classes more likely represent heteroplasmy within a single parasite than within a subpopulation of cells. Furthermore, the presence of SNPs <3 bp apart on contiguous sequence reads may have non-synonymous coding implications, although their relative rarity, and a lack of indels suggest that minority and majority maxicircle variants would not differ phenotypically. Finally, the presence of heteroplasmy at less than 0.5% of sites indicates it is unlikely to represent a major source of typing error when using maxicircle Sanger sequencing to characterize isolates.

Several factors are likely to contribute to mitochondrial heteroplasmy. Mutation in length or nucleotide composition and/or bi-parental inheritance in genetic exchange events are both exacerbated by differential replication rates and inequitable cytoplasmic segregation of mitochondrial genomes during mitosis [50], [51]. In kinetoplastids, maxicircle intra-clone diversity in the non-coding region was previously reported in both *T. cruzi* [31] and *Leishmania major* [52], [53]. In addition, an earlier study attributed a change in *T. cruzi* maxicircle gene repertoire (elimination of one of two heteroplasmic *ND7* amplicons) to sub-culture [54]. However, biologically cloned samples were not used and the possibility of a mixed infection was excluded on the basis of only four microsatellite loci. Sylvio X10/1 (a biological clone produced by micromanipulation) was first isolated from a Brazilian patient in 1979 [55] and has been in intermittent sub-culture ever since. The retention of minor maxicircle classes in Sylvio X10/1 for over thirty years suggests that a heteroplasmic state in *T. cruzi* is naturally sustained.

The observations that *T. cruzi* mitochondrial heteroplasmy is not present at sufficient levels to adversely disrupt phylogenetic reconstructions stimulated the development of the mtMLST scheme and its assessment against traditional nuclear targets. Initially, three types of nuclear marker were evaluated, each characterized by different rates of evolution. Unsurprisingly *GPI* was highly conserved across TcI and lacked sufficient resolution to discriminate between isolates. The slow accumulation of point mutations at housekeeping loci, which are generally under purifying selection, renders these targets more appropriate to describe inter-DTU variation. Thus they are valuable candidates for inclusion in traditional nuclear MLST schemes [56]. The mini-exon SL-IR is widely used as a TcI taxonomic marker in view of its heterogeneity and ease of amplification [57]. In this study, SL-IR variability manifested as a ten-fold increase in sequence diversity as compared to that of *GPI*, and supported the robust delineation of two nuclear populations (VEN_silv_ and ARG_North_). However, there are several caveats associated with the SL-IR, notably the presence of multiple tandemly-repeated copies with undefined chromosomal orthology between strains [58]. Previous attempts to estimate the level of intra-isolate SL-IR diversity have reported >96% homology between copies [19]. However, only ten clones were sequenced from each sample, representing less than 10% of the ∼200 copies present per genome. Recent observations of substantial variation in gene copy number and chromosomal arrangement between *T. cruzi* strains further discourage the use of such targets for taxonomy [59]. In addition, numerous indels in the SL-IR prevent the sequencing of a suitable outgroup [39] and multiple ambiguous alignments, introduced by the microsatellite region, can disrupt phylogenetic signals [60]. Ultimately both *GPI* and the SL-IR suffer from the same fundamental criticism that single genes are inadequate to infer the overall phylogeny of an entire species [61]. Recombination, gene conversion and concerted evolution have all contributed to the genealogical history of *T. cruzi* [62] but remain undetectable using single loci.

The 25 microsatellite loci afforded the highest level of resolution from an individual set of markers, defining three statistically-supported groupings (VEN_dom_, ARG_North_ and ANDES_Bol/Chile_). Their superior performance compared to *GPI* and the SL-IR is expected considering microsatellites are neutrally-evolving, co-dominant and hypervariable with mutation rates several orders of magnitude higher than protein-coding genes [63]. However, the use of these markers is not devoid of limitations. Most importantly, microsatellites are particularly sensitive to homoplasy, a situation where two alleles are identical in sequence but not descent, and thus fail to discriminate between closely related but evolutionarily distinct strains [64]. The three nuclear markers (*GPI*, SL-IR and microsatellites) were concatenated based on the assumption that no robust incongruence was observed between individual phylogenetic trees. However, concatenating these data did not have a significant additive effect on the level of resolution, with just three populations (VEN_silv_, ARG_North_ and ANDES_Bol/Chile_) emerging as well-supported groups. Importantly this dataset did reveal a subdivision in the BOL_North_ group, which went undetected by all individual nuclear markers.

Gross incongruence between the mtMLST and nuclear phylogenies revealed two incidences of inter-DTU mitochondrial introgression, indicative of multiple genetic exchange events in *T. cruzi*. Introgression was detected in North America, where identical maxicircles were observed in sylvatic TcI and TcIV isolates. A 1.25 kb fragment (*COII-ND1*) of this TcIV maxicircle haplotype has been previously described in other TcI samples from the US states of Georgia and Florida [11], [27]. On the basis of the limited nuclear loci examined, and in line with previous work [27], only TcI derived nuclear genetic material appears to have been retained in these hybrids. The genetic disparity between North and South American TcIV isolates, coupled with their geographical and ecological isolation [65], implies that this event most likely occurred in North/Central America. A second, independent novel mitochondrial introgression event was identified in a Venezuelan clinical isolate. This TcI strain (9354) shares its maxicircle haplotype with a subset of human and sylvatic TcIV and TcIII isolates from Bolivia, Venezuela and Colombia, consistent with a local and possibly recent origin. Presumably TcIV, a known secondary agent of human Chagas disease in Venezuela, is a more likely donor candidate than TcIII, which is largely absent from domestic transmission cycles [4].

Nonetheless, evidence of homogeneous maxicircle sequences in multiple, geographically dispersed isolates from different transmission cycles implies the occurrence of several genetic exchange events. It is conceivable that the TcIV/TcIII-type maxicircle sampled in this study is a relic from a TcI antecedent, supporting a common ancestry between TcI, TcIII and TcIV [9]. Alternatively, this haplotype may have originated from a TcIV or TcIII strain and its distribution reflects a recent unidirectional backcrossing event into TcI. Introgression is a more parsimonious explanation than the retention of ancestral polymorphisms through incomplete lineage sorting, particularly in areas of sympatry or parapatry among DTUs [66]. However, the historical diversification of TcI [67] and TcIII [68]–[70], driven by disparate ecological niches [71], and the current separation between most arboreal and terrestrial transmission cycles of TcIV and TcIII, respectively, challenge the likelihood of secondary contact between these lineages, a prerequisite of introgressive hybridization. Resolving the donor DTU of this event is complicated by the presence of indistinguishable mitochondrial sequences and paradoxically divergent nuclear genes in TcIII and TcIV isolates. It is unclear whether this results from a mechanism acting to homogenize maxicircles while allowing nuclear genes to slowly deviate [11] (unlikely), repeated and recurrent backcrossing (more likely), or merely reflects the relative paucity of available TcIV and TcIII genotypes for comparison (a certainty).

Regardless of the underlying mechanisms, it is clear that genetic exchange continues to influence the natural population structure of *T. cruzi* TcI. In this study, the failure to detect reciprocal transfer of nuclear DNA using an array of loci readily demonstrates the importance of adopting an integrative approach, complementing traditional nuclear markers with multiple mitochondrial targets. In the absence of comparative genomics, it is impossible to establish whether mitochondrial introgression is entirely independent of nuclear recombination.

Another advantage of the mtMLST scheme is its ability to reveal cryptic sub-DTU diversity. The significantly different evolutionary histories of the nuclear and maxicircle genes from members of BOL_North_ and ARG_North_ are consistent with intra-lineage recombination. The low levels of diversity observed within this incongruent maxicircle clade are indicative of recent and possibly multiple exchange events. In addition, two divergent maxicircles from AM_North/Cen_ have also exposed a level of diversity that conflicts with earlier reports of reduced genetic differentiation in this group resulting from their recent biogeographical expansion [18], [72]. Furthermore, the incongruent basal phylogenetic position of most of BRAZ_North-East_ in the maxicircle tree as well as the presence of another divergent maxicircle in one isolate (IM48) from this population highlights the extent to which intra-lineage diversity can be neglected by other genotyping methods. The phylogenetic placement of IM48 suggests it may be the product of an intra-TcI introgression event. However, IM48 is also a geographical outlier within the BRAZ_North-East_ population and it is difficult to determine the origin of this maxicircle haplotype in the absence of additional isolates from West-Central Amazonia.

The mechanisms governing maxicircle genetic exchange and the origins of heteroplasmy observed in Sylvio X10/1 are debatable. Currently, all reported maxicircle inheritance in natural [11] and experimental *T. cruzi* hybrids [24] is uniparental. However, the demonstration of heteroplasmy in this study suggests that, following genetic exchange, any minor maxicircle genotypes may be undetectable using conventional sequencing techniques. In addition, evidence of bi-parental transmission of both maxicircles [73], [74] and minicircles [75] in experimentally-derived *T. brucei* hybrids indicates that this phenomenon can occur in kinetoplastids as a result of recombination. The mechanism of genetic exchange in *T. cruzi* [24] differs from meiosis, which is observed in *T. brucei* [73], [76]. Current data suggest *in vitro* recombination in *T. cruzi* may be analogous to the parasexual cycle of *Candida albicans* where nuclear fusion creates a tetraploid intermediate, followed by genome erosion and reversion to aneuploidy [24], [77], [78]. It is not implausible to suggest that the process of cell fusion and nuclear re-assortment may be accompanied by asymmetrical kinetoplast distribution to progeny cells. Furthermore, the sequence redundancy observed among minicircle guide RNAs has been postulated to allow biparental inheritance to occur with no detrimental consequences to mitochondrial RNA editing and hybrid viability [79].

Most importantly, the phenotypic implications of mitochondrial heteroplasmy and introgression in *T. cruzi* are unknown. Maxicircles play a fundamental role in parasite metabolism and development in the triatomine bug vector. Therefore the relationship between genetic recombination and phenotypic heterogeneity may have important implications for disease epidemiology. mtMLST presents a valuable new strategy to detect directional gene flow and examine the dispersal history of *T. cruzi* at the transmission cycle level. Furthermore, mtMLST is an excellent tool to identify genetic exchange between closely related isolates in conjunction with nuclear MLMT data. By adopting a combined nuclear and mitochondrial approach, one can simultaneously address local, epidemiologically important hypotheses as well as robustly identify parasite mating systems. Thus in combination with adequate spatio-temporal sampling, we strongly recommend this methodology as an alternative to exclusively nuclear or mitochondrial population genetic studies in future work with medically important trypanosomes. Finally, the level of resolution that the mtMLST method provides should greatly facilitate attempts to elucidate the relationship between specific parasite genotypes and phenotypic traits relating to Chagas disease pathology.

## Supporting Information

Table S1 **Panel of reference strains from the six** ***T. cruzi*** **DTUs.**
(DOCX)

Table S2 **Additional** ***T. cruzi*** **TcIII and TcIV isolates used in selected analyses.**
(DOCX)

Table S3 **Microsatellite loci and primer sequences.**
(DOCX)

Table S4 **Heteroplasmic sites in the Sylvio X10/1 maxicircle genome.**
(DOCX)

Dataset S1 **Concatenated nuclear dataset spreadsheet.** Individual Neighbour-Joining trees were constructed for both nuclear genes (SL-IR and *GPI*) and the 25 microsatellite loci. Once all trees were visualized independently to confirm congruent topologies, nuclear SNPs were re-coded numerically and concatenated with microsatellite data in this spreadsheet. *D*_AS_ values were calculated for this concatenated dataset and used to generate a single Neighbour-Joining tree encompassing all nuclear genetic diversity.
(XLSX)
