# Supplementary material for: Multiple Mitochondrial Introgression Events and Heteroplasmy in Trypanosoma cruzi Revealed by Maxicircle MLST and Next Generation Sequencing
Source: PLoS Negl Trop Dis. 2012 Apr 10;6(4):e1584. doi: 10.1371/journal.pntd.0001584 (PMC3323513; doi:10.1371/journal.pntd.0001584)
Supplement: Table S1 — Panel of reference strains from the six T. cruzi DTUs. (DOCX) [file pntd.0001584.s001.docx]

**Table S1.** Panel of reference strains from the six *T. cruzi* DTUs

| **Strain** | **DTU** | **Location** | **Host/Vector** |
| --- | --- | --- | --- |
| Sylvio X10/1 | TcI | Belém, Brazil | *Homo sapiens* |
| Esm cl3 | TcII | Sáo Felipe, Brazil | *Homo sapiens* |
| M5631 cl5 | TcIII | Marajo, Para, Brazil | *Dasypus novemcinctus* |
| CanIII cl1 | TcIV | Belém, Brazil | *Homo sapiens* |
| Sc43 cl1 | TcV | Santa Cruz, Bolivia | *Triatoma infestans* |
| CL Brener | TcVI | Rio Grande Do Sul, Brazil | *Triatoma infestans* |
