# Supplementary material for: Multiple Mitochondrial Introgression Events and Heteroplasmy in Trypanosoma cruzi Revealed by Maxicircle MLST and Next Generation Sequencing
Source: PLoS Negl Trop Dis. 2012 Apr 10;6(4):e1584. doi: 10.1371/journal.pntd.0001584 (PMC3323513; doi:10.1371/journal.pntd.0001584)
Supplement: Table S2 — Additional T. cruzi TcIII and TcIV isolates used in selected analyses. (DOCX) [file pntd.0001584.s002.docx]

**Table S2.** Additional *T. cruzi* TcIII and TcIV isolates used in selected analyses

| **Strain Code** | **Strain** | **DTU** | **Location** | **Host/Vector** |
| --- | --- | --- | --- | --- |
| 92122 | 92122102R | TcIV | Georgia, USA | *Procyon lotor* |
| Can3 | CanIII cl1 | TcIV | Belém, Brazil | *Homo sapiens* |
| ERA | ERA cl2 | TcIV | Anzoátegui, Venezuela | *Homo sapiens* |
| 10R26 | 10R26 | TcIV | Santa Cruz, Bolivia | *Aotus* Sp. |
| Sairi3 | Saimiri3 cl1 | TcIV | Venezuela | *Saimiri sciureus* |
| X106 | X10610 cl5 | TcIV | Guárico, Venezuela | *Homo sapiens* |
| CM17 | CM17 | TcIII | Carimaga, Colombia | *Dasypus* |
