# Supplementary material for: Multiple Mitochondrial Introgression Events and Heteroplasmy in Trypanosoma cruzi Revealed by Maxicircle MLST and Next Generation Sequencing
Source: PLoS Negl Trop Dis. 2012 Apr 10;6(4):e1584. doi: 10.1371/journal.pntd.0001584 (PMC3323513; doi:10.1371/journal.pntd.0001584)
Supplement: Table S3 — Microsatellite loci and primer sequences. (DOCX) [file pntd.0001584.s003.docx]

**Table S3.** Microsatellite loci and primer sequences

| **Chromosome^a^** | **Primer code** | **Repeat type** | **Forward/Reverse Primer (5’-3’)** |
| --- | --- | --- | --- |
| 6 | 6529(CA)a | (CA)_n_ | TGTGAAATGATTTGACCCGA |
|  |  |  | AGAGTCACGCCGCAAAGTAT |
| 6 | 6529(TA)b | (TA)_n_ | TGAAGGAGATTCTCTGCGGT |
|  |  |  | CTCTCATCTTTTGTTGTGTCCG |
| 6 | mclf10 | (CA)_n_A(CA)_n_ | GCGTAGCGATTCATTTCC |
|  |  |  | ATCCGCTACCACTATCCAC |
| 10 | 6855(TA)(GA) | (TA)_n_(GA)_n_ | TGTGATCAACGCGCATAAAT |
|  |  |  | TTCCATTGCCTCGTTTTAGA |
| 15 | 11863(CA) | (CA)_n_ | AGTTGACATCCCCAAGCAAG |
|  |  |  | CCCTGATGCTGCAGACTCTT |
| 19 | TcUn3 | Unknown | CTTAAAGAGATACAAGAGGGAAGG |
|  |  |  | CTGTTATTTCAATAACACGGGG |
| 19 | 10101(TA) | (TA)_n_ | AACCCGCGCAGATACATTAG |
|  |  |  | TTCATTTGCAGCAACACACA |
| 24 | 8741(TA) | (TA)_n_ | TGTAACGGTAGGTCTCAATTCG |
|  |  |  | TTGCACTTGTGTATCTCGCC |
| 27 | 10101(TC) | (TC)_n_ | CGTACGACGTGGACACAAAC |
|  |  |  | ACAAGTGGGTGAGCCAAAAG |
| 27 | 10101(CA)c | (CA)_n_ | GTGTCGTTGCTCCCAAACTC |
|  |  |  | AAACTTGCCAAATGTGAGGG |
| 27 | 10101(CA)a | (CA)_n_ | GTCGCCATCATGTACAAACG |
|  |  |  | CTGTTGGCGAATGGTCATAA |
| 34 | 6559(TC) | (TC)_n_ | CGCTCTCAAAGGCACCTTAC |
|  |  |  | ATATGGACGCGTAGGAGTGC |
| 37 | 10187(TTA) | (TTA)_n_ | GAGAGAGATTCGGAAACTAATAGC |
|  |  |  | CATGTCCCTTCCTCCGTAAA |
| 37 | 10187(CA)(TA) | (CA)_n_(TA)_n_ | CATGTCATTAAGTGGCCACG |
|  |  |  | GCACATGTTGGTTGTTGGAA |
| 37 | 10187(TA) | (TA)_n_ | AGAAAAAGGTTTACAACGAGCG |
|  |  |  | CGATGGAGAACGTGAAACAA |
| 37 | 10187(GA) | (GA)_n_ | GTCACACCACTAGCGATGACA |
|  |  |  | ACTGCACAATACCCCCTTTG |
| 37 | TcUn2 | Unknown | AACAAAATCTAGCGTCTACCATCC |
|  |  |  | GGTGTTGGCGTGTATGATTG |
| 39 | 6925(TG)b | (TG)_n_ | GAAACGCACTCACCCACAC |
|  |  |  | GGTAGCAACGCCAAACTTTC |
| 39 | 7093(TC) | (TC)_n_ | CCAACATTCAACAAGGGAAA |
|  |  |  | GCATGAATATTGCCGGATCT |
| 39 | 6925(CT) | (CT)_n_ | CATCAAGGAAAAACGGAGGA |
|  |  |  | CGGTACCACCTCAAGGAAAG |
| 39 | 7093(TA)c | (TA)_n_ | CGTGTGCACAGGAGAGAAAA |
|  |  |  | CGTTTGGAGGAGGATTGAGA |
| 39 | 6925(TG)a | (TG)_n_ | TCGTTCTCTTTACGCTTGCA |
|  |  |  | TAGCAGCACCAAACAAAACG |
| 39 | 7093(TCC) | (TCC)_n_ | AGACGTTCATATTCGCAGCC |
|  |  |  | AGCCACATCCACATTTCCTC |
| 40 | 11283(TCG) | (TCG)_n_ | ACCACCAGGAGGACATGAAG |
|  |  |  | TGTACACGGAACAGCGAAG |
| 40 | 11283(TA)b | (TA)_n_ | AACATCCTCCACCTCACAGG |
|  |  |  | TTTGAATGCGAGGTGGTACA |

^a^ Chromosomal assignment based on Weatherly *et al.,* 2009 [40]
