# Supplementary material for: Multiple Mitochondrial Introgression Events and Heteroplasmy in Trypanosoma cruzi Revealed by Maxicircle MLST and Next Generation Sequencing
Source: PLoS Negl Trop Dis. 2012 Apr 10;6(4):e1584. doi: 10.1371/journal.pntd.0001584 (PMC3323513; doi:10.1371/journal.pntd.0001584)
Supplement: Table S4 — Heteroplasmic sites in the Sylvio X10/1 maxicircle genome. (DOCX) [file pntd.0001584.s004.docx]

**Table S4.** Heteroplasmic sites in the Sylvio X10/1 maxicircle genome

| **Coding Region** | **Position** | **Ref. Base** | **Total Read Depth** | **Read Depth By Base^a^** | | | |
| --- | --- | --- | --- | --- | --- | --- | --- |
|  |  |  |  | A | C | G | T |
| ***12S rRNA*** | 269 | A | 223 | 204 | **-** | **19** | - |
| ***12S rRNA*** | 283 | A | 169 | 158 | - | - | **11** |
| ***12S rRNA*** | 369 | A | 126 | 120 | - | - | **6** |
| ***12S rRNA*** | 371 | T | 138 | - | - | **6** | 132 |
| ***12S rRNA*** | 377 | C | 166 | **6** | 160 | - | - |
| ***12S rRNA*** | 380 | A | 175 | 169 | **6** | - | **-** |
| ***12S rRNA*** | 381 | T | 179 | **6** | - | - | 173 |
| ***12S rRNA*** | 387 | A | 199 | 192 | - | **7** | - |
| ***12S rRNA*** | 390 | A | 207 | 201 | - | **6** | - |
| ***12S rRNA*** | 400 | C | 212 | **6** | 206 | **-** | - |
| **Intergenic** | 1190 | T | 208 | **13** | - | - | 195 |
| **Intergenic** | 1195 | T | 201 | **-** | **13** | - | 188 |
| **Intergenic** | 1196 | T | 192 | - | **13** | - | 179 |
| ***9S rRNA*** | 1205 | G | 183 | **-** | - | 170 | **13** |
| ***9S rRNA*** | 1209 | A | 172 | 159 | - | **-** | **13** |
| ***9S rRNA*** | 1239 | A | 110 | 105 | - | **5** | **-** |
| ***9S rRNA*** | 1244 | T | 114 | - | - | **5** | 109 |
| ***9S rRNA*** | 1671 | G | 186 | **10** | - | 176 | - |
| ***9S rRNA*** | 1688 | A | 170 | 160 | - | **10** | - |
| ***9S rRNA*** | 1798 | A | 44 | 28 | - | - | **16** |
| **Intergenic** | 1844 | A | 49 | 43 | - | **6** | - |
| ***MURF5*** | 2555 | G | 180 | - | - | 116 | **64** |
| ***MURF5*** | 2567 | A | 260 | 179 | - | **81** | **-** |
| ***MURF5*** | 2576 | A | 179 | 133 | - | - | **46** |
| ***MURF5*** | 2578 | A | 141 | 107 | - | - | **34** |
| ***CYT b*** | 5230 | G | 248 | **6** | **-** | 242 | - |
| ***MURF1*** | 6436 | A | 182 | 167 | - | **15** | **-** |
| ***MURF1*** | 6442 | C | 171 | **-** | 156 | - | **15** |
| ***MURF1*** | 6618 | A | 72 | 57 | - | - | **15** |
| ***MURF1*** | 6620 | G | 73 | **-** | - | 58 | **15** |
| ***MURF1*** | 6736 | G | 59 | **12** | - | 47 | - |
| ***MURF1*** | 6748 | A | 52 | 39 | - | - | **13** |
| ***MURF1*** | 6965 | G | 268 | - | **7** | 261 | **-** |
| ***MURF2*** | 8998 | A | 59 | 54 | - | **5** | **-** |
| ***MURF2*** | 9144 | T | 129 | - | - | **23** | 106 |
| ***MURF2*** | 9145 | A | 132 | 110 | **-** | - | **22** |
| ***MURF2*** | 9147 | A | 134 | 112 | - | **22** | **-** |
| ***MURF2*** | 9150 | T | 135 | **22** | - | - | 113 |
| ***MURF2*** | 9156 | G | 150 | - | - | 128 | **22** |
| ***MURF2*** | 9162 | T | 155 | - | - | **23** | 132 |
| ***MURF2*** | 9168 | A | 179 | 157 | **22** | - | **-** |
| ***MURF2*** | 9621 | T | 76 | **18** | - | - | 58 |
| ***MURF2*** | 9622 | T | 76 | **21** | - | - | 55 |
| ***MURF2*** | 9627 | T | 76 | - | - | **7** | 69 |
| ***MURF2*** | 9628 | T | 76 | - | **7** | - | 69 |
| ***MURF2*** | 9629 | A | 77 | 60 | **17** | - | **-** |
| ***MURF2*** | 9630 | A | 78 | 60 | **18** | - | **-** |
| ***MURF2*** | 9631 | T | 81 | - | **7** | - | 74 |
| ***MURF2*** | 9632 | C | 83 | **9** | 74 | - | **-** |
| ***MURF2*** | 9637 | T | 89 | - | - | **7** | 82 |
| ***MURF2*** | 9639 | A | 91 | 57 | - | - | **34** |
| ***MURF2*** | 9646 | G | 61 | - | **14** | 47 | **-** |
| ***MURF2*** | 9655 | G | 82 | - | - | 68 | **14** |
| ***CR4*** | 11520 | T | 257 | **18** | - | - | 239 |
| ***CR4*** | 11525 | C | 262 | - | 244 | - | **18** |
| ***CR4*** | 11535 | C | 269 | **21** | 248 | - | **-** |
| ***CR4*** | 11544 | T | 282 | **21** | - | - | 261 |
| ***CR4*** | 11563 | A | 349 | 328 | - | - | **21** |
| **Intergenic** | 11785 | T | 155 | **6** | - | - | 149 |
| ***ND4*** | 11987 | C | 229 | **-** | 170 | - | **59** |
| ***ND4*** | 11999 | A | 229 | 170 | - | - | **59** |
| ***ND4*** | 12001 | A | 229 | 171 | - | - | **58** |
| ***ND4*** | 12038 | C | 113 | **8** | 105 | - | - |
| ***ND4*** | 12043 | A | 120 | 111 | - | **9** | - |
| ***ND4*** | 12044 | C | 127 | **-** | 118 | - | **9** |
| ***ND4*** | 12045 | A | 126 | 115 | - | **11** | - |
| ***ND4*** | 12046 | T | 132 | **-** | - | **8** | 124 |
| ***ND4*** | 12051 | A | 146 | 138 | - | - | **8** |
| ***ND4*** | 12054 | T | 154 | **8** | - | - | 146 |
| ***ND4*** | 12070 | C | 227 | **-** | 219 | **8** | - |
| ***ND4*** | 12081 | A | 248 | 240 | - | **8** | - |
| ***ND4*** | 12101 | T | 361 | **32** | - | - | 329 |
| ***ND4*** | 12104 | G | 370 | **-** | - | 338 | **32** |
| ***ND4*** | 12105 | A | 374 | 342 | **32** | - | - |

^a^ Minor base displayed in bold
